# Tris(benzyl­diphenyl­phosphane-κ*P*)(nitrato-κ*O*)silver(I)

**DOI:** 10.1107/S2414314622011476

**Published:** 2022-12-06

**Authors:** Kariska Potgieter, Frederick P. Malan, Oyekunle Azeez Alimi, Reinout Meijboom

**Affiliations:** a Department of Chemical Sciences, University of Johannesburg, PO Box 524, Auckland Park, 2006, Johannesburg, South Africa; b Department of Chemistry, University of Pretoria, Lynnwood Road, Hatfield, Pretoria, 0002, South Africa; Vienna University of Technology, Austria

**Keywords:** crystal structure, silver(I) phosphine inorganic complex

## Abstract

The mol­ecular structure of the title complex, [Ag(NO_3_)(C_19_H_17_P)_3_], exhibits a severely distorted tetra­hedral coordination environment around the central Ag^I^ atom, comprising one O and three P atoms. Apart from a primary Ag—O coordination of the nitrato ligand of 2.667 (3) Å, a second (weaker) secondary inter­action of the nitrato ligand *via* the other O atom of 3.118 (4) Å is observed. The compound crystallizes with a complete mol­ecule in the asymmetric unit. Weak C—H⋯O inter­actions consolidate the packing.

## Abstract 2

The central Ag^I^ atom exhibits a distorted tetra­hedral coordination environment, defined by three P atoms of three benzyl-di­phenyl­phosphane ligands and one O atom of a nitrato ligand.

## Graphical abstract

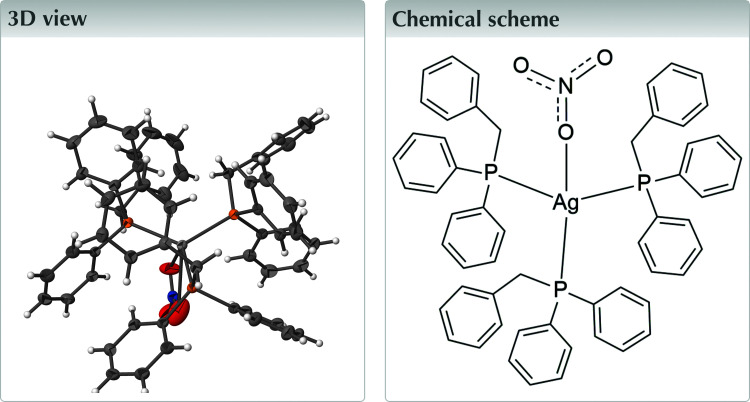

## Structure description

As the class of bioactive silver(I) phosphine complexes continues to be a research focus area of several research groups (Potgieter *et al.*, 2017 ▸), the unpredictability of the solid-state mol­ecular structures of these compounds remain inter­esting and is continuously studied by means of X-ray diffraction techniques (Potgieter *et al.*, 2022 ▸).

**Figure 1:**
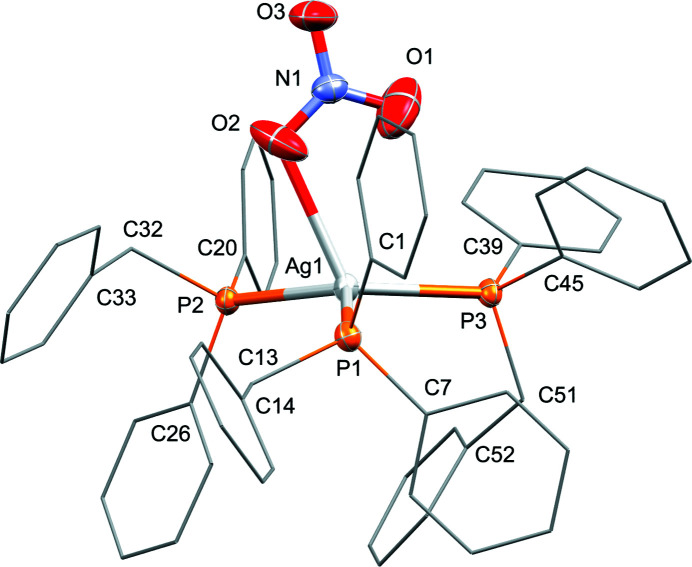
Mol­ecular structure of the silver(I) tris-phosphine complex. Displacement ellipsoids are drawn at the 50% probability level. For clarity, selected carbon atoms of the arene rings are shown in wireframe style and hydrogen atoms are omitted.

The mol­ecular structure of the title compound is shown in Fig. 1 ▸. The complex crystallizes with a complete mol­ecule in the asymmetric unit. The central Ag^I^ atom features three coordinating benzyl-di­phenyl­phosphine ligands, as well as one nitrato ligand. An umbrella configuration is seen with the three phosphino ligands on top of a plane with the central Ag^I^ atom at the apex with a plane-to-Ag separation of *ca* 0.361 Å. The near symmetric binding of the phosphine ligands are indicated by the narrow range of Ag—P bond lengths between 2.4737 (5) and 2.4990 (6) Å, which are within the known range of related Ag^I^ phosphine compounds (Meijboom *et al.*, 2009 ▸). The distorted tetra­hedral coordination environment displayed by the Ag^I^ cation is underpinned by the corresponding bond angles P1—Ag1—P2 (123.727 (19)°), P1—Ag1—P3 (106.310 (19)°), and P2—Ag1—P3 (123.634 (19)°). The nitrato coordinates to the Ag^I^ atom *via* O2 (Ag1—O2 = 2.667 (5) Å). A secondary weak inter­action between O1 and Ag1 is also observed, with an inter­action distance of 3.118 (4) Å, which is thought to help stabilize the coordination around Ag1. The three N—O bond lengths of the nitrato ligand are nearly identical with a range between 1.221 (3) and 1.233 (3) Å. The NO_3_ ligand and the Ag1—P3 bond all lie (almost) within the same plane, with P1 and P2 on either side of the plane. Corresponding torsion angles are N1—O2—Ag1—P1 = 110.78 (18)°, N1—O2—Ag1—P2 = −127.26 (19)°, and N1—O2—Ag1—P3 = −5.9 (2)°. The concentration of bulky arene groups from the three phosphine ligands also does not appear to notably affect the tetra­hedral environment of each of the P atoms, with typical C′—P—C′′ bond angles between 99.02 (10)–105.80 (11)°, and an average of 103.40°.

**Figure 2:**
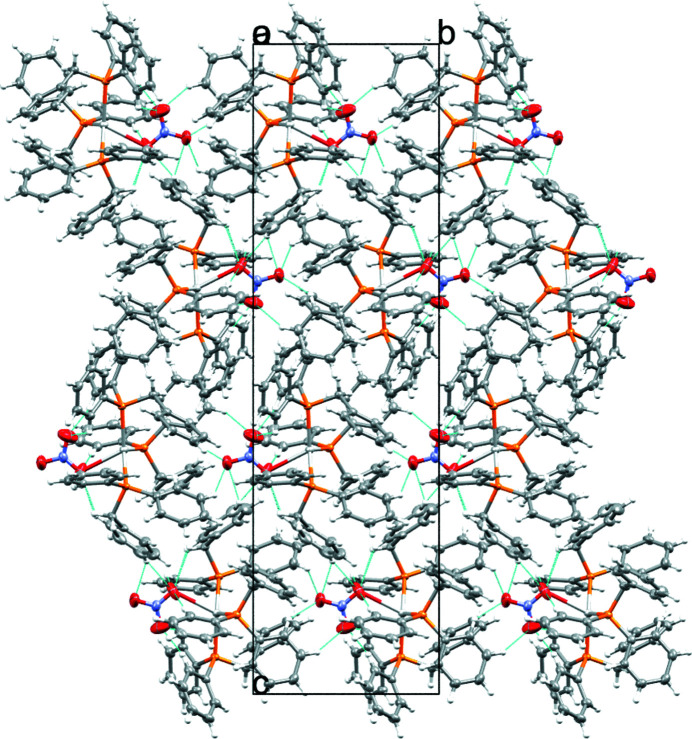
Packing diagram as viewed along the *a* axis of the structure of the title compound. Non-classical hydrogen-bonding inter­actions are indicated with cyan dotted lines.

The complex packs in three dimensions as isolated layers of complexes featuring a metal-containing NO_3_ rich layer and an alternating arene-rich layer (Fig. 2 ▸). No classical hydrogen-bonding or mentionable π–π stacking inter­actions are observed. Selected non-classical intra- and inter-mol­ecular hydrogen bonding inter­actions (C—H⋯O) are shown in Fig. 2 ▸ and included in Table 1 ▸.

## Synthesis and crystallization

Benzyl­diphenyl­phosphine (3 mmol) was added to a solution of silver nitrate (1 mmol) in 20 ml aceto­nitrile. The reaction mixture was heated under reflux for a few hours. It was filtered and left to form crystals. Small colourless crystals were obtained overnight.

## Refinement

Crystal data, data collection and structure refinement details are summarized in Table 2 ▸.

## Supplementary Material

Crystal structure: contains datablock(s) I. DOI: 10.1107/S2414314622011476/wm4175sup1.cif

Structure factors: contains datablock(s) I. DOI: 10.1107/S2414314622011476/wm4175Isup2.hkl

Supporting information file. DOI: 10.1107/S2414314622011476/wm4175Isup3.cdx

CCDC reference: 2223250

Additional supporting information:  crystallographic information; 3D view; checkCIF report

## Figures and Tables

**Table 1:** Hydrogen-bond geometry (Å, °)

*D*—H⋯*A*	*D*—H	H⋯*A*	*D*⋯*A*	*D*—H⋯*A*
C2—H2⋯O2	0.95	2.51	3.4612 (4)	175
C8—H8⋯O3^i^	0.95	2.33	3.102 (4)	139
C28—H28⋯O3^i^	0.95	2.30	3.096 (4)	141
C46—H46⋯O1	0.95	2.44	3.231 (4)	141

Symmetry code: (i) 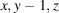 .

**Table 2:** Experimental details

Crystal data	
Chemical formula	[Ag(NO_3_)(C_19_H_17_P)_3_]
*M*_r_	998.76
Crystal system, space group	Monoclinic, *P*2_1_/*c*
Temperature (K)	150
*a*, *b*, *c* (Å)	13.5643 (2), 9.9979 (2), 35.4391 (6)
β (°)	99.248 (2)
*V* (Å^3^)	4743.59 (15)
*Z*	4
Radiation type	Mo *K*α
μ (mm^−1^)	0.57
Crystal size (mm)	0.21 × 0.18 × 0.16

Data collection	
Diffractometer	XtaLAB Synergy R, DW system, HyPix
Absorption correction	Multi-scan (*CrysAlis PRO*; Rigaku OD, 2022 ▸)
*T*_min_, *T*_max_	0.269, 1.000
No. of measured, independent and observed [*I* > 2σ(*I*)] reflections	77751, 12895, 10926
*R*_int_	0.051
(sin θ/λ)_max_ (Å^−1^)	0.727

Refinement	
*R*[*F* ^2^ > 2σ(*F* ^2^)], *wR*(*F* ^2^), *S*	0.040, 0.096, 1.07
No. of reflections	12895
No. of parameters	586
H-atom treatment	H-atom parameters constrained
Δρ_max_, Δρ_min_ (e Å^−3^)	0.90, −0.78

Computer programs: *CrysAlis PRO* (Rigaku OD, 2022 ▸), *SHELXT* (Sheldrick, 2015*a* ▸), *SHELXL* (Sheldrick, 2015*b* ▸) and *OLEX2* (Dolomanov *et al.*, 2009 ▸).

## full crystallographic data

### Crystal data

**Table:**

[Ag(NO_3_)(C_19_H_17_P)_3_]	*F*(000) = 2064
*M**_r_* = 998.76	*D*_x_ = 1.399 Mg m^−^^3^
Monoclinic, *P*2_1_/*c*	Mo *K*α radiation, λ = 0.71073 Å
*a* = 13.5643 (2) Å	Cell parameters from 49121 reflections
*b* = 9.9979 (2) Å	θ = 2.6–31.3°
*c* = 35.4391 (6) Å	µ = 0.57 mm^−^^1^
β = 99.248 (2)°	*T* = 150 K
*V* = 4743.59 (15) Å^3^	Block, colourless
*Z* = 4	0.21 × 0.18 × 0.16 mm

### Data collection

**Table:**

XtaLAB Synergy R, DW system, HyPix diffractometer	12895 independent reflections
Radiation source: Rotating-anode X-ray tube, Rigaku (Mo) X-ray Source	10926 reflections with *I* > 2σ(*I*)
Mirror monochromator	*R*_int_ = 0.051
Detector resolution: 10.0000 pixels mm^-1^	θ_max_ = 31.1°, θ_min_ = 2.4°
ω scans	*h* = −18→15
Absorption correction: multi-scan (CrysAlisPro; Rigaku OD, 2022)	*k* = −14→14
*T*_min_ = 0.269, *T*_max_ = 1.000	*l* = −43→49
77751 measured reflections	

### Refinement

**Table:**

Refinement on *F*^2^	Primary atom site location: dual
Least-squares matrix: full	Hydrogen site location: inferred from neighbouring sites
*R*[*F*^2^ > 2σ(*F*^2^)] = 0.040	H-atom parameters constrained
*wR*(*F*^2^) = 0.096	*w* = 1/[σ^2^(*F*_o_^2^) + (0.0341*P*)^2^ + 5.9508*P*] where *P* = (*F*_o_^2^ + 2*F*_c_^2^)/3
*S* = 1.07	(Δ/σ)_max_ = 0.002
12895 reflections	Δρ_max_ = 0.90 e Å^−^^3^
586 parameters	Δρ_min_ = −0.78 e Å^−^^3^
0 restraints	

### Special details

**Table:**

Geometry. All esds (except the esd in the dihedral angle between two l.s. planes) are estimated using the full covariance matrix. The cell esds are taken into account individually in the estimation of esds in distances, angles and torsion angles; correlations between esds in cell parameters are only used when they are defined by crystal symmetry. An approximate (isotropic) treatment of cell esds is used for estimating esds involving l.s. planes.

### Fractional atomic coordinates and isotropic or equivalent isotropic displacement parameters (Å^2^)

**Table:**

	*x*	*y*	*z*	*U*_iso_*/*U*_eq_	
Ag1	0.74798 (2)	0.69605 (2)	0.37571 (2)	0.02091 (5)	
P1	0.58242 (4)	0.59000 (6)	0.37839 (2)	0.02200 (11)	
P2	0.83407 (4)	0.68482 (5)	0.31933 (2)	0.02064 (11)	
P3	0.83722 (4)	0.70570 (5)	0.44299 (2)	0.02071 (11)	
N1	0.72422 (15)	1.0224 (2)	0.36449 (6)	0.0296 (4)	
C7	0.59306 (15)	0.4655 (2)	0.41680 (6)	0.0229 (4)	
O2	0.70309 (18)	0.9312 (2)	0.34174 (8)	0.0674 (8)	
C45	0.75566 (16)	0.7562 (2)	0.47712 (6)	0.0235 (4)	
O3	0.7075 (3)	1.1395 (2)	0.35565 (8)	0.0745 (8)	
C52	0.93733 (16)	0.4638 (2)	0.43405 (6)	0.0234 (4)	
C20	0.95104 (16)	0.7790 (2)	0.32598 (6)	0.0229 (4)	
C39	0.95066 (15)	0.8048 (2)	0.45606 (6)	0.0218 (4)	
C26	0.86546 (17)	0.5186 (2)	0.30363 (6)	0.0244 (4)	
C40	0.98711 (17)	0.8780 (2)	0.42795 (7)	0.0268 (5)	
H40	0.9504	0.8811	0.4028	0.032*	
C8	0.62320 (17)	0.3353 (2)	0.41041 (7)	0.0288 (5)	
H8	0.6300	0.3083	0.3853	0.035*	
C12	0.58538 (17)	0.5036 (2)	0.45431 (6)	0.0257 (4)	
H12	0.5659	0.5922	0.4594	0.031*	
C32	0.77378 (17)	0.7596 (2)	0.27341 (6)	0.0263 (4)	
H32A	0.7634	0.8561	0.2776	0.032*	
H32B	0.8207	0.7520	0.2548	0.032*	
C27	0.8137 (2)	0.4102 (2)	0.31479 (7)	0.0326 (5)	
H27	0.7700	0.4221	0.3330	0.039*	
C51	0.87716 (17)	0.5366 (2)	0.46029 (6)	0.0251 (4)	
H51A	0.9180	0.5442	0.4860	0.030*	
H51B	0.8172	0.4828	0.4628	0.030*	
C1	0.48491 (16)	0.7039 (2)	0.38875 (6)	0.0243 (4)	
C25	1.04431 (18)	0.7184 (2)	0.33519 (7)	0.0287 (5)	
H25	1.0486	0.6241	0.3382	0.034*	
C13	0.52699 (17)	0.5003 (2)	0.33411 (6)	0.0279 (5)	
H13A	0.5334	0.5586	0.3120	0.034*	
H13B	0.5677	0.4193	0.3318	0.034*	
C33	0.67541 (18)	0.6995 (2)	0.25573 (6)	0.0288 (5)	
C14	0.41952 (17)	0.4579 (2)	0.33067 (6)	0.0265 (5)	
C9	0.64343 (18)	0.2446 (3)	0.44029 (8)	0.0334 (5)	
H9	0.6632	0.1560	0.4354	0.040*	
C21	0.94684 (18)	0.9193 (2)	0.32359 (7)	0.0273 (5)	
H21	0.8839	0.9630	0.3185	0.033*	
C53	1.03062 (17)	0.5100 (2)	0.42783 (7)	0.0283 (5)	
H53	1.0562	0.5917	0.4391	0.034*	
C57	0.90097 (18)	0.3451 (2)	0.41677 (7)	0.0290 (5)	
H57	0.8365	0.3145	0.4199	0.035*	
C54	1.08633 (18)	0.4372 (2)	0.40518 (7)	0.0300 (5)	
H54	1.1489	0.4704	0.4005	0.036*	
C44	1.00465 (17)	0.8037 (2)	0.49331 (6)	0.0268 (4)	
H44	0.9797	0.7556	0.5129	0.032*	
C22	1.03420 (19)	0.9945 (3)	0.32863 (7)	0.0330 (5)	
H22	1.0306	1.0892	0.3266	0.040*	
C31	0.93097 (18)	0.4982 (3)	0.27725 (7)	0.0301 (5)	
H31	0.9665	0.5718	0.2691	0.036*	
C6	0.40306 (18)	0.6613 (3)	0.40516 (7)	0.0307 (5)	
H6	0.3940	0.5689	0.4098	0.037*	
C46	0.67781 (17)	0.8448 (2)	0.46439 (7)	0.0288 (5)	
H46	0.6711	0.8810	0.4393	0.035*	
C19	0.39218 (19)	0.3394 (3)	0.34654 (8)	0.0339 (5)	
H19	0.4421	0.2833	0.3603	0.041*	
C50	0.76469 (19)	0.7058 (3)	0.51408 (7)	0.0315 (5)	
H50	0.8178	0.6463	0.5232	0.038*	
C41	1.07705 (19)	0.9469 (3)	0.43651 (7)	0.0321 (5)	
H41	1.1016	0.9966	0.4171	0.039*	
C5	0.33482 (18)	0.7550 (3)	0.41466 (7)	0.0363 (6)	
H5	0.2800	0.7264	0.4263	0.044*	
C56	0.9573 (2)	0.2707 (3)	0.39504 (8)	0.0347 (5)	
H56	0.9321	0.1886	0.3840	0.042*	
C2	0.49522 (19)	0.8392 (2)	0.38089 (7)	0.0311 (5)	
H2	0.5499	0.8687	0.3693	0.037*	
C42	1.13090 (18)	0.9434 (3)	0.47317 (7)	0.0323 (5)	
H42	1.1928	0.9893	0.4788	0.039*	
O1	0.7673 (3)	0.9985 (4)	0.39716 (8)	0.0922 (11)	
C55	1.0505 (2)	0.3162 (3)	0.38947 (7)	0.0336 (5)	
H55	1.0896	0.2646	0.3749	0.040*	
C38	0.5872 (2)	0.7659 (3)	0.25900 (8)	0.0385 (6)	
H38	0.5893	0.8438	0.2745	0.046*	
C11	0.60614 (19)	0.4122 (3)	0.48413 (7)	0.0325 (5)	
H11	0.6005	0.4388	0.5094	0.039*	
C43	1.09414 (18)	0.8724 (3)	0.50165 (7)	0.0306 (5)	
H43	1.1305	0.8711	0.5269	0.037*	
C10	0.63498 (19)	0.2827 (3)	0.47725 (8)	0.0341 (5)	
H10	0.6488	0.2206	0.4977	0.041*	
C23	1.1262 (2)	0.9324 (3)	0.33650 (7)	0.0360 (6)	
H23	1.1857	0.9839	0.3395	0.043*	
C28	0.8251 (2)	0.2829 (3)	0.29945 (9)	0.0432 (7)	
H28	0.7878	0.2094	0.3066	0.052*	
C48	0.61863 (19)	0.8284 (3)	0.52465 (8)	0.0360 (6)	
H48	0.5714	0.8520	0.5406	0.043*	
C24	1.13108 (19)	0.7946 (3)	0.34007 (8)	0.0351 (5)	
H24	1.1942	0.7518	0.3459	0.042*	
C15	0.3448 (2)	0.5380 (3)	0.31089 (8)	0.0361 (5)	
H15	0.3619	0.6201	0.3001	0.043*	
C47	0.61029 (19)	0.8801 (3)	0.48816 (8)	0.0348 (6)	
H47	0.5577	0.9406	0.4792	0.042*	
C30	0.9440 (2)	0.3715 (3)	0.26309 (7)	0.0371 (6)	
H30	0.9899	0.3580	0.2458	0.045*	
C49	0.6962 (2)	0.7419 (3)	0.53789 (8)	0.0367 (6)	
H49	0.7029	0.7072	0.5631	0.044*	
C29	0.8905 (2)	0.2646 (3)	0.27398 (8)	0.0413 (6)	
H29	0.8988	0.1782	0.2638	0.050*	
C18	0.2920 (2)	0.3019 (3)	0.34248 (8)	0.0437 (7)	
H18	0.2741	0.2207	0.3536	0.052*	
C17	0.2190 (2)	0.3817 (3)	0.32255 (8)	0.0416 (7)	
H17	0.1508	0.3556	0.3198	0.050*	
C34	0.6704 (2)	0.5834 (3)	0.23413 (8)	0.0424 (6)	
H34	0.7296	0.5353	0.2320	0.051*	
C16	0.2450 (2)	0.4988 (3)	0.30674 (8)	0.0412 (6)	
H16	0.1947	0.5538	0.2928	0.049*	
C4	0.3467 (2)	0.8895 (3)	0.40719 (8)	0.0411 (6)	
H4	0.3004	0.9529	0.4140	0.049*	
C3	0.4257 (2)	0.9316 (3)	0.38990 (9)	0.0420 (6)	
H3	0.4326	1.0236	0.3842	0.050*	
C37	0.4957 (2)	0.7200 (4)	0.23997 (10)	0.0534 (9)	
H37	0.4361	0.7674	0.2420	0.064*	
C36	0.4922 (3)	0.6057 (4)	0.21824 (10)	0.0627 (11)	
H36	0.4300	0.5741	0.2051	0.075*	
C35	0.5782 (3)	0.5373 (4)	0.21551 (10)	0.0596 (9)	
H35	0.5750	0.4576	0.2008	0.072*	

### Atomic displacement parameters (Å^2^)

**Table:**

	*U*^11^	*U*^22^	*U*^33^	*U*^12^	*U*^13^	*U*^23^
Ag1	0.02052 (8)	0.02433 (8)	0.01770 (8)	−0.00022 (6)	0.00252 (6)	−0.00111 (6)
P1	0.0190 (2)	0.0257 (3)	0.0212 (3)	−0.0010 (2)	0.0029 (2)	−0.0022 (2)
P2	0.0229 (3)	0.0230 (2)	0.0163 (2)	−0.0005 (2)	0.0038 (2)	0.00008 (19)
P3	0.0196 (2)	0.0239 (3)	0.0179 (2)	0.0023 (2)	0.0008 (2)	−0.00154 (19)
N1	0.0258 (10)	0.0267 (10)	0.0377 (11)	0.0026 (8)	0.0092 (8)	0.0034 (8)
C7	0.0170 (9)	0.0257 (10)	0.0257 (11)	−0.0009 (8)	0.0026 (8)	−0.0004 (8)
O2	0.0477 (13)	0.0483 (13)	0.101 (2)	0.0064 (10)	−0.0031 (13)	−0.0391 (14)
C45	0.0199 (10)	0.0271 (10)	0.0231 (10)	−0.0001 (8)	0.0022 (8)	−0.0054 (8)
O3	0.132 (3)	0.0289 (11)	0.0673 (17)	0.0155 (13)	0.0303 (17)	0.0030 (11)
C52	0.0233 (10)	0.0249 (10)	0.0215 (10)	0.0057 (8)	0.0016 (8)	0.0030 (8)
C20	0.0261 (10)	0.0261 (10)	0.0163 (9)	−0.0024 (8)	0.0035 (8)	−0.0016 (8)
C39	0.0201 (9)	0.0243 (10)	0.0202 (10)	0.0026 (8)	0.0009 (8)	−0.0034 (8)
C26	0.0289 (11)	0.0243 (10)	0.0198 (10)	0.0026 (8)	0.0032 (9)	−0.0011 (8)
C40	0.0278 (11)	0.0309 (11)	0.0216 (10)	−0.0017 (9)	0.0035 (9)	−0.0036 (8)
C8	0.0253 (11)	0.0294 (11)	0.0319 (12)	0.0038 (9)	0.0047 (9)	−0.0036 (9)
C12	0.0265 (11)	0.0263 (10)	0.0251 (11)	0.0013 (8)	0.0064 (9)	−0.0007 (8)
C32	0.0292 (11)	0.0270 (10)	0.0208 (10)	−0.0039 (9)	−0.0016 (9)	0.0039 (8)
C27	0.0378 (13)	0.0281 (11)	0.0336 (13)	0.0018 (10)	0.0112 (11)	0.0028 (10)
C51	0.0261 (11)	0.0251 (10)	0.0244 (11)	0.0042 (8)	0.0054 (9)	0.0018 (8)
C1	0.0211 (10)	0.0298 (11)	0.0203 (10)	0.0020 (8)	−0.0015 (8)	−0.0028 (8)
C25	0.0319 (12)	0.0285 (11)	0.0241 (11)	0.0013 (9)	−0.0002 (9)	0.0018 (9)
C13	0.0256 (11)	0.0364 (12)	0.0220 (11)	−0.0042 (9)	0.0045 (9)	−0.0052 (9)
C33	0.0321 (12)	0.0321 (11)	0.0204 (10)	−0.0074 (10)	−0.0016 (9)	0.0062 (9)
C14	0.0249 (11)	0.0336 (11)	0.0206 (10)	−0.0029 (9)	0.0021 (9)	−0.0083 (9)
C9	0.0280 (12)	0.0276 (11)	0.0445 (15)	0.0063 (9)	0.0056 (11)	0.0021 (10)
C21	0.0295 (11)	0.0271 (11)	0.0253 (11)	−0.0005 (9)	0.0045 (9)	−0.0029 (9)
C53	0.0269 (11)	0.0261 (11)	0.0323 (12)	0.0032 (9)	0.0064 (9)	−0.0004 (9)
C57	0.0259 (11)	0.0304 (11)	0.0303 (12)	0.0016 (9)	0.0039 (9)	−0.0003 (9)
C54	0.0269 (11)	0.0325 (12)	0.0319 (12)	0.0044 (9)	0.0091 (10)	0.0022 (9)
C44	0.0262 (11)	0.0320 (11)	0.0206 (10)	0.0010 (9)	−0.0013 (9)	−0.0001 (9)
C22	0.0386 (13)	0.0282 (11)	0.0308 (12)	−0.0072 (10)	0.0013 (10)	−0.0041 (9)
C31	0.0328 (12)	0.0347 (12)	0.0242 (11)	0.0016 (10)	0.0085 (10)	−0.0015 (9)
C6	0.0248 (11)	0.0384 (13)	0.0280 (12)	0.0010 (9)	0.0014 (9)	−0.0044 (10)
C46	0.0246 (11)	0.0304 (11)	0.0307 (12)	0.0010 (9)	0.0018 (9)	−0.0068 (9)
C19	0.0297 (12)	0.0360 (13)	0.0340 (13)	−0.0053 (10)	−0.0013 (10)	−0.0021 (10)
C50	0.0306 (12)	0.0373 (13)	0.0271 (12)	0.0053 (10)	0.0061 (10)	−0.0019 (10)
C41	0.0325 (12)	0.0332 (12)	0.0321 (12)	−0.0068 (10)	0.0097 (10)	−0.0040 (10)
C5	0.0225 (11)	0.0558 (16)	0.0297 (13)	0.0063 (11)	0.0019 (10)	−0.0082 (11)
C56	0.0387 (14)	0.0307 (12)	0.0345 (13)	0.0004 (10)	0.0049 (11)	−0.0082 (10)
C2	0.0287 (12)	0.0317 (12)	0.0308 (12)	0.0023 (9)	−0.0015 (10)	0.0010 (9)
C42	0.0230 (11)	0.0339 (12)	0.0390 (14)	−0.0036 (9)	0.0021 (10)	−0.0116 (10)
O1	0.096 (2)	0.124 (3)	0.0568 (17)	0.056 (2)	0.0128 (15)	0.0268 (17)
C55	0.0366 (13)	0.0346 (12)	0.0313 (12)	0.0087 (10)	0.0105 (10)	−0.0038 (10)
C38	0.0350 (13)	0.0405 (14)	0.0372 (14)	−0.0014 (11)	−0.0023 (11)	0.0130 (11)
C11	0.0313 (12)	0.0390 (13)	0.0283 (12)	−0.0006 (10)	0.0077 (10)	0.0034 (10)
C43	0.0265 (11)	0.0352 (12)	0.0274 (12)	0.0017 (9)	−0.0037 (9)	−0.0068 (9)
C10	0.0316 (12)	0.0327 (12)	0.0382 (14)	0.0004 (10)	0.0061 (11)	0.0099 (10)
C23	0.0322 (13)	0.0427 (14)	0.0311 (13)	−0.0116 (11)	−0.0011 (10)	−0.0059 (10)
C28	0.0555 (17)	0.0247 (12)	0.0497 (17)	−0.0021 (11)	0.0098 (14)	0.0010 (11)
C48	0.0286 (12)	0.0435 (14)	0.0386 (14)	0.0001 (10)	0.0138 (11)	−0.0157 (11)
C24	0.0258 (11)	0.0430 (14)	0.0336 (13)	0.0009 (10)	−0.0038 (10)	−0.0051 (11)
C15	0.0351 (13)	0.0389 (13)	0.0328 (13)	0.0000 (11)	0.0011 (11)	−0.0019 (10)
C47	0.0260 (12)	0.0377 (13)	0.0397 (14)	0.0069 (10)	0.0027 (10)	−0.0116 (11)
C30	0.0429 (15)	0.0431 (14)	0.0258 (12)	0.0108 (12)	0.0072 (11)	−0.0071 (10)
C49	0.0386 (14)	0.0458 (15)	0.0275 (12)	0.0015 (11)	0.0110 (11)	−0.0050 (11)
C29	0.0523 (17)	0.0302 (12)	0.0388 (14)	0.0109 (12)	−0.0004 (13)	−0.0085 (11)
C18	0.0374 (15)	0.0515 (17)	0.0417 (15)	−0.0168 (13)	0.0048 (12)	−0.0027 (13)
C17	0.0250 (12)	0.0670 (19)	0.0322 (13)	−0.0090 (12)	0.0033 (10)	−0.0140 (13)
C34	0.0514 (17)	0.0417 (15)	0.0312 (13)	−0.0112 (13)	−0.0026 (12)	−0.0037 (11)
C16	0.0288 (13)	0.0565 (17)	0.0359 (14)	0.0073 (12)	−0.0023 (11)	−0.0072 (12)
C4	0.0325 (13)	0.0478 (16)	0.0394 (15)	0.0154 (12)	−0.0053 (11)	−0.0121 (12)
C3	0.0410 (15)	0.0342 (13)	0.0469 (16)	0.0105 (11)	−0.0044 (12)	−0.0029 (11)
C37	0.0331 (14)	0.065 (2)	0.056 (2)	−0.0113 (14)	−0.0099 (14)	0.0251 (16)
C36	0.051 (2)	0.073 (2)	0.053 (2)	−0.0326 (18)	−0.0236 (16)	0.0225 (17)
C35	0.075 (2)	0.0534 (19)	0.0436 (18)	−0.0279 (18)	−0.0112 (17)	−0.0057 (14)

### Geometric parameters (Å, º)

**Table:**

Ag1—P1	2.4990 (6)	C54—H54	0.9500
Ag1—P2	2.4737 (5)	C44—C43	1.385 (3)
Ag1—P3	2.4964 (6)	C44—H44	0.9500
Ag1—O2	2.667 (3)	C22—C23	1.381 (4)
Ag1—O1	3.118 (4)	C22—H22	0.9500
P1—C1	1.827 (2)	C31—C30	1.384 (4)
P1—C7	1.833 (2)	C31—H31	0.9500
P1—C13	1.858 (2)	C6—C5	1.395 (3)
P2—C26	1.824 (2)	C6—H6	0.9500
P2—C20	1.827 (2)	C46—C47	1.386 (3)
P2—C32	1.857 (2)	C46—H46	0.9500
P3—C39	1.826 (2)	C19—C18	1.395 (4)
P3—C45	1.836 (2)	C19—H19	0.9500
P3—C51	1.850 (2)	C50—C49	1.399 (3)
N1—O2	1.221 (3)	C50—H50	0.9500
N1—O3	1.224 (3)	C41—C42	1.385 (4)
N1—O1	1.233 (3)	C41—H41	0.9500
C7—C8	1.394 (3)	C5—C4	1.385 (4)
C7—C12	1.403 (3)	C5—H5	0.9500
C45—C50	1.390 (3)	C56—C55	1.386 (4)
C45—C46	1.396 (3)	C56—H56	0.9500
C52—C57	1.389 (3)	C2—C3	1.393 (4)
C52—C53	1.397 (3)	C2—H2	0.9500
C52—C51	1.518 (3)	C42—C43	1.390 (4)
C20—C25	1.393 (3)	C42—H42	0.9500
C20—C21	1.407 (3)	C55—H55	0.9500
C39—C40	1.390 (3)	C38—C37	1.391 (4)
C39—C44	1.403 (3)	C38—H38	0.9500
C26—C27	1.383 (3)	C11—C10	1.385 (4)
C26—C31	1.404 (3)	C11—H11	0.9500
C40—C41	1.391 (3)	C43—H43	0.9500
C40—H40	0.9500	C10—H10	0.9500
C8—C9	1.388 (4)	C23—C24	1.384 (4)
C8—H8	0.9500	C23—H23	0.9500
C12—C11	1.391 (3)	C28—C29	1.375 (4)
C12—H12	0.9500	C28—H28	0.9500
C32—C33	1.505 (3)	C48—C47	1.381 (4)
C32—H32A	0.9900	C48—C49	1.384 (4)
C32—H32B	0.9900	C48—H48	0.9500
C27—C28	1.402 (4)	C24—H24	0.9500
C27—H27	0.9500	C15—C16	1.395 (4)
C51—H51A	0.9900	C15—H15	0.9500
C51—H51B	0.9900	C47—H47	0.9500
C1—C2	1.393 (3)	C30—C29	1.381 (4)
C1—C6	1.400 (3)	C30—H30	0.9500
C25—C24	1.389 (3)	C49—H49	0.9500
C25—H25	0.9500	C29—H29	0.9500
C13—C14	1.504 (3)	C18—C17	1.376 (4)
C13—H13A	0.9900	C18—H18	0.9500
C13—H13B	0.9900	C17—C16	1.369 (4)
C33—C34	1.386 (4)	C17—H17	0.9500
C33—C38	1.390 (4)	C34—C35	1.395 (4)
C14—C19	1.388 (4)	C34—H34	0.9500
C14—C15	1.389 (4)	C16—H16	0.9500
C9—C10	1.387 (4)	C4—C3	1.383 (4)
C9—H9	0.9500	C4—H4	0.9500
C21—C22	1.390 (3)	C3—H3	0.9500
C21—H21	0.9500	C37—C36	1.375 (6)
C53—C54	1.393 (3)	C37—H37	0.9500
C53—H53	0.9500	C36—C35	1.368 (6)
C57—C56	1.385 (3)	C36—H36	0.9500
C57—H57	0.9500	C35—H35	0.9500
C54—C55	1.387 (4)		
			
P2—Ag1—P3	123.634 (19)	C23—C22—C21	120.4 (2)
P2—Ag1—P1	123.727 (19)	C23—C22—H22	119.8
P3—Ag1—P1	106.310 (19)	C21—C22—H22	119.8
C1—P1—C7	104.31 (10)	C30—C31—C26	120.3 (2)
C1—P1—C13	105.23 (10)	C30—C31—H31	119.8
C7—P1—C13	105.80 (11)	C26—C31—H31	119.8
C1—P1—Ag1	115.30 (8)	C5—C6—C1	119.8 (2)
C7—P1—Ag1	110.64 (7)	C5—C6—H6	120.1
C13—P1—Ag1	114.63 (7)	C1—C6—H6	120.1
C26—P2—C20	105.56 (10)	C47—C46—C45	120.3 (2)
C26—P2—C32	101.02 (10)	C47—C46—H46	119.8
C20—P2—C32	99.02 (10)	C45—C46—H46	119.8
C26—P2—Ag1	116.82 (7)	C14—C19—C18	120.5 (3)
C20—P2—Ag1	112.65 (7)	C14—C19—H19	119.8
C32—P2—Ag1	119.40 (8)	C18—C19—H19	119.8
C39—P3—C45	104.83 (10)	C45—C50—C49	120.5 (2)
C39—P3—C51	102.82 (10)	C45—C50—H50	119.7
C45—P3—C51	102.03 (10)	C49—C50—H50	119.7
C39—P3—Ag1	121.52 (7)	C42—C41—C40	120.2 (2)
C45—P3—Ag1	112.88 (7)	C42—C41—H41	119.9
C51—P3—Ag1	110.63 (8)	C40—C41—H41	119.9
O2—N1—O3	122.1 (3)	C4—C5—C6	120.2 (3)
O2—N1—O1	120.1 (3)	C4—C5—H5	119.9
O3—N1—O1	117.8 (3)	C6—C5—H5	119.9
C8—C7—C12	118.4 (2)	C57—C56—C55	120.0 (2)
C8—C7—P1	120.24 (17)	C57—C56—H56	120.0
C12—C7—P1	120.70 (17)	C55—C56—H56	120.0
C50—C45—C46	118.8 (2)	C1—C2—C3	120.3 (3)
C50—C45—P3	123.34 (17)	C1—C2—H2	119.9
C46—C45—P3	117.77 (17)	C3—C2—H2	119.9
C57—C52—C53	118.6 (2)	C41—C42—C43	119.9 (2)
C57—C52—C51	119.4 (2)	C41—C42—H42	120.1
C53—C52—C51	121.9 (2)	C43—C42—H42	120.1
C25—C20—C21	118.3 (2)	C56—C55—C54	119.9 (2)
C25—C20—P2	122.93 (17)	C56—C55—H55	120.1
C21—C20—P2	118.70 (17)	C54—C55—H55	120.1
C40—C39—C44	119.1 (2)	C33—C38—C37	121.0 (3)
C40—C39—P3	119.10 (16)	C33—C38—H38	119.5
C44—C39—P3	121.72 (17)	C37—C38—H38	119.5
C27—C26—C31	118.8 (2)	C10—C11—C12	120.6 (2)
C27—C26—P2	118.20 (17)	C10—C11—H11	119.7
C31—C26—P2	122.59 (18)	C12—C11—H11	119.7
C39—C40—C41	120.3 (2)	C44—C43—C42	120.2 (2)
C39—C40—H40	119.8	C44—C43—H43	119.9
C41—C40—H40	119.8	C42—C43—H43	119.9
C9—C8—C7	120.9 (2)	C11—C10—C9	119.4 (2)
C9—C8—H8	119.6	C11—C10—H10	120.3
C7—C8—H8	119.6	C9—C10—H10	120.3
C11—C12—C7	120.3 (2)	C22—C23—C24	119.7 (2)
C11—C12—H12	119.8	C22—C23—H23	120.2
C7—C12—H12	119.8	C24—C23—H23	120.2
C33—C32—P2	116.14 (16)	C29—C28—C27	119.8 (3)
C33—C32—H32A	108.3	C29—C28—H28	120.1
P2—C32—H32A	108.3	C27—C28—H28	120.1
C33—C32—H32B	108.3	C47—C48—C49	119.7 (2)
P2—C32—H32B	108.3	C47—C48—H48	120.2
H32A—C32—H32B	107.4	C49—C48—H48	120.2
C26—C27—C28	120.5 (2)	C23—C24—C25	120.4 (2)
C26—C27—H27	119.7	C23—C24—H24	119.8
C28—C27—H27	119.7	C25—C24—H24	119.8
C52—C51—P3	113.11 (15)	C14—C15—C16	120.6 (3)
C52—C51—H51A	109.0	C14—C15—H15	119.7
P3—C51—H51A	109.0	C16—C15—H15	119.7
C52—C51—H51B	109.0	C48—C47—C46	120.7 (2)
P3—C51—H51B	109.0	C48—C47—H47	119.6
H51A—C51—H51B	107.8	C46—C47—H47	119.6
C2—C1—C6	119.4 (2)	C29—C30—C31	120.2 (2)
C2—C1—P1	117.77 (18)	C29—C30—H30	119.9
C6—C1—P1	122.84 (18)	C31—C30—H30	119.9
C24—C25—C20	120.7 (2)	C48—C49—C50	119.9 (2)
C24—C25—H25	119.6	C48—C49—H49	120.0
C20—C25—H25	119.6	C50—C49—H49	120.0
C14—C13—P1	117.38 (16)	C28—C29—C30	120.3 (2)
C14—C13—H13A	108.0	C28—C29—H29	119.9
P1—C13—H13A	108.0	C30—C29—H29	119.9
C14—C13—H13B	108.0	C17—C18—C19	120.4 (3)
P1—C13—H13B	108.0	C17—C18—H18	119.8
H13A—C13—H13B	107.2	C19—C18—H18	119.8
C34—C33—C38	118.7 (2)	C16—C17—C18	119.6 (3)
C34—C33—C32	121.7 (2)	C16—C17—H17	120.2
C38—C33—C32	119.5 (2)	C18—C17—H17	120.2
C19—C14—C15	118.4 (2)	C33—C34—C35	119.9 (3)
C19—C14—C13	121.8 (2)	C33—C34—H34	120.0
C15—C14—C13	119.8 (2)	C35—C34—H34	120.0
C10—C9—C8	120.3 (2)	C17—C16—C15	120.5 (3)
C10—C9—H9	119.8	C17—C16—H16	119.8
C8—C9—H9	119.8	C15—C16—H16	119.8
C22—C21—C20	120.4 (2)	C3—C4—C5	120.2 (2)
C22—C21—H21	119.8	C3—C4—H4	119.9
C20—C21—H21	119.8	C5—C4—H4	119.9
C54—C53—C52	120.5 (2)	C4—C3—C2	120.1 (3)
C54—C53—H53	119.8	C4—C3—H3	120.0
C52—C53—H53	119.8	C2—C3—H3	120.0
C56—C57—C52	121.0 (2)	C36—C37—C38	119.6 (3)
C56—C57—H57	119.5	C36—C37—H37	120.2
C52—C57—H57	119.5	C38—C37—H37	120.2
C55—C54—C53	120.0 (2)	C35—C36—C37	120.1 (3)
C55—C54—H54	120.0	C35—C36—H36	119.9
C53—C54—H54	120.0	C37—C36—H36	119.9
C43—C44—C39	120.3 (2)	C36—C35—C34	120.7 (3)
C43—C44—H44	119.9	C36—C35—H35	119.6
C39—C44—H44	119.9	C34—C35—H35	119.6
			
C1—P1—C7—C8	150.85 (18)	C25—C20—C21—C22	2.9 (3)
C13—P1—C7—C8	40.1 (2)	P2—C20—C21—C22	179.77 (18)
Ag1—P1—C7—C8	−84.58 (18)	C57—C52—C53—C54	−0.9 (3)
C1—P1—C7—C12	−38.5 (2)	C51—C52—C53—C54	176.6 (2)
C13—P1—C7—C12	−149.21 (18)	C53—C52—C57—C56	2.6 (3)
Ag1—P1—C7—C12	86.08 (18)	C51—C52—C57—C56	−175.0 (2)
C39—P3—C45—C50	−81.0 (2)	C52—C53—C54—C55	−1.7 (4)
C51—P3—C45—C50	25.9 (2)	C40—C39—C44—C43	−1.4 (3)
Ag1—P3—C45—C50	144.65 (18)	P3—C39—C44—C43	175.40 (18)
C39—P3—C45—C46	102.23 (19)	C20—C21—C22—C23	−0.8 (4)
C51—P3—C45—C46	−150.84 (18)	C27—C26—C31—C30	−0.7 (4)
Ag1—P3—C45—C46	−32.1 (2)	P2—C26—C31—C30	−172.94 (19)
C26—P2—C20—C25	−27.4 (2)	C2—C1—C6—C5	−2.2 (3)
C32—P2—C20—C25	−131.61 (19)	P1—C1—C6—C5	175.73 (18)
Ag1—P2—C20—C25	101.16 (18)	C50—C45—C46—C47	−0.8 (4)
C26—P2—C20—C21	155.82 (18)	P3—C45—C46—C47	176.11 (19)
C32—P2—C20—C21	51.63 (19)	C15—C14—C19—C18	0.3 (4)
Ag1—P2—C20—C21	−75.59 (18)	C13—C14—C19—C18	−179.2 (2)
C45—P3—C39—C40	−128.35 (18)	C46—C45—C50—C49	0.8 (4)
C51—P3—C39—C40	125.31 (18)	P3—C45—C50—C49	−175.9 (2)
Ag1—P3—C39—C40	1.0 (2)	C39—C40—C41—C42	−0.2 (4)
C45—P3—C39—C44	54.8 (2)	C1—C6—C5—C4	1.3 (4)
C51—P3—C39—C44	−51.5 (2)	C52—C57—C56—C55	−1.6 (4)
Ag1—P3—C39—C44	−175.81 (15)	C6—C1—C2—C3	1.1 (4)
C20—P2—C26—C27	146.77 (19)	P1—C1—C2—C3	−176.9 (2)
C32—P2—C26—C27	−110.5 (2)	C40—C41—C42—C43	−1.0 (4)
Ag1—P2—C26—C27	20.7 (2)	C57—C56—C55—C54	−1.0 (4)
C20—P2—C26—C31	−40.9 (2)	C53—C54—C55—C56	2.7 (4)
C32—P2—C26—C31	61.8 (2)	C34—C33—C38—C37	−2.2 (4)
Ag1—P2—C26—C31	−167.02 (17)	C32—C33—C38—C37	173.8 (2)
C44—C39—C40—C41	1.4 (3)	C7—C12—C11—C10	0.2 (4)
P3—C39—C40—C41	−175.52 (18)	C39—C44—C43—C42	0.2 (4)
C12—C7—C8—C9	1.2 (3)	C41—C42—C43—C44	1.0 (4)
P1—C7—C8—C9	172.02 (18)	C12—C11—C10—C9	0.2 (4)
C8—C7—C12—C11	−0.9 (3)	C8—C9—C10—C11	0.0 (4)
P1—C7—C12—C11	−171.74 (18)	C21—C22—C23—C24	−1.2 (4)
C26—P2—C32—C33	67.3 (2)	C26—C27—C28—C29	1.9 (4)
C20—P2—C32—C33	175.23 (19)	C22—C23—C24—C25	1.0 (4)
Ag1—P2—C32—C33	−62.3 (2)	C20—C25—C24—C23	1.1 (4)
C31—C26—C27—C28	−1.2 (4)	C19—C14—C15—C16	−0.8 (4)
P2—C26—C27—C28	171.4 (2)	C13—C14—C15—C16	178.6 (2)
C57—C52—C51—P3	−118.1 (2)	C49—C48—C47—C46	1.0 (4)
C53—C52—C51—P3	64.4 (3)	C45—C46—C47—C48	−0.1 (4)
C39—P3—C51—C52	−78.03 (18)	C26—C31—C30—C29	1.9 (4)
C45—P3—C51—C52	173.49 (16)	C47—C48—C49—C50	−1.0 (4)
Ag1—P3—C51—C52	53.16 (17)	C45—C50—C49—C48	0.1 (4)
C7—P1—C1—C2	144.10 (18)	C27—C28—C29—C30	−0.8 (4)
C13—P1—C1—C2	−104.76 (19)	C31—C30—C29—C28	−1.1 (4)
Ag1—P1—C1—C2	22.6 (2)	C14—C19—C18—C17	0.3 (4)
C7—P1—C1—C6	−33.9 (2)	C19—C18—C17—C16	−0.2 (4)
C13—P1—C1—C6	77.3 (2)	C38—C33—C34—C35	1.5 (4)
Ag1—P1—C1—C6	−155.40 (17)	C32—C33—C34—C35	−174.5 (3)
C21—C20—C25—C24	−3.0 (3)	C18—C17—C16—C15	−0.4 (4)
P2—C20—C25—C24	−179.79 (19)	C14—C15—C16—C17	0.9 (4)
C1—P1—C13—C14	−40.4 (2)	C6—C5—C4—C3	0.7 (4)
C7—P1—C13—C14	69.7 (2)	C5—C4—C3—C2	−1.8 (4)
Ag1—P1—C13—C14	−168.14 (16)	C1—C2—C3—C4	0.9 (4)
P2—C32—C33—C34	−81.8 (3)	C33—C38—C37—C36	1.4 (4)
P2—C32—C33—C38	102.3 (2)	C38—C37—C36—C35	0.3 (5)
P1—C13—C14—C19	−84.4 (3)	C37—C36—C35—C34	−1.0 (5)
P1—C13—C14—C15	96.2 (2)	C33—C34—C35—C36	0.1 (5)
C7—C8—C9—C10	−0.7 (4)		

### Hydrogen-bond geometry (Å, º)

**Table:**

*D*—H···*A*	*D*—H	H···*A*	*D*···*A*	*D*—H···*A*
C2—H2···O2	0.95	2.51	3.4612 (4)	175
C8—H8···O3^i^	0.95	2.33	3.102 (4)	139
C28—H28···O3^i^	0.95	2.30	3.096 (4)	141
C46—H46···O1	0.95	2.44	3.231 (4)	141

Symmetry code: (i) *x*, *y*−1, *z*.
